# Implementing of aMMP‐8 point‐of‐care test with a modified new disease classification in Finnish adolescent cohorts

**DOI:** 10.1002/cre2.603

**Published:** 2022-06-08

**Authors:** Anna M. Heikkinen, Teija Raivisto, Ismo Räisänen, Taina Tervahartiala, Nagihan Bostanci, Timo Sorsa

**Affiliations:** ^1^ Department of Oral and Maxillofacial Diseases and Public Health University of Helsinki and Helsinki University Hospital Helsinki Finland; ^2^ Department of Dental Medicine Karolinska Institutet Stockholm Sweden; ^3^ Faculty of Medicine and Health Technology University of Tampere Tampere Finland; ^4^ Special Oral Health Care Unit Tampere Finland; ^5^ Hameenlinna Kaupunki Hämeenlinna Finland; ^6^ Department of Oral and Maxillofacial Diseases, Head and Neck Center University of Helsinki and Helsinki University Hospital Helsinki Finland

**Keywords:** adolescent, gingivitis, matrix metalloproteinase 8, periodontitis

## Abstract

**Objectives:**

Periodontitis is a multifactorial biofilm‐induced inflammatory disease; however, clinical and radiographic information reflects events that have already occurred. aMMP‐8 lateral‐flow chairside or point‐of‐care‐test (POC‐test) results have also come to complement the overall status of the patient's current situation. The current study aimed to clarify the usefulness of aMMP‐8 chairside‐test (aMMP‐8 POCT) results to alert the initial or early periodontitis in adolescents, a potential periodontitis risk group with cut off 20 ng/ml in Stage I periodontitis, according to the current periodontitis classification system.

**Material and Methods:**

A total of 117 adolescents were aMMP‐8 POC tested for aMMP‐8 levels and examined for the full mouth and were interviewed for questions concerning health behavior.

**Results:**

Of all 117 participants, *N* = 38 (32.5%) was aMMP‐8 POCT positive, and *N* = 34 (29.1%) had at least one 4 mm periodontal deepened pocket; however, only *N* = 16 (13.7%) had both characteristics. The subclinical stage (*N* = 47) could not be classified either as gingivitis or Stage I. Of the participants, who did not have any deepened periodontal pockets, 18  tested negative.

**Conclusions:**

Stage I is preceded by antecedent stages that should be tackled by oral healthcare prevention and personalized treatment modalities by professionals. Elevated (>20 ng/ml), positive aMMP‐8 POCT results can be regarded as initial alarmer such as emerging risk. This should be utilized in the preventive personalized interventions by oral health professionals.

## 1 INTRODUCTION

Periodontitis is the most common global chronic inflammatory infectious disease (Ximenez‐Fyvie et al., 2000), and 75% of adults suffer from periodontal disease in Finland (Suominen et al., 2018). Of all adolescents, 10%–15% (depending on criteria) suffer from an early stage of periodontitis in Finland (Heikkinen et al., 2008). Periodontitis is a multifactorial biofilm‐induced inflammatory disease (Hajishengallis, 2020). It has been addressed that systemic diseases such as diabetes (Preshaw et al., 2012; Tonetti et al., 2017), most strongly evidenced, and coronary heart (Tiensripojamarn et al., 2021) disease are associated with periodontitis. Types 1 and 2 diabetes mellitus, as well as periodontitis, are interlinked to elevated levels of systemic inflammatory markers, enhancing each other's influence (Preshaw et al., 2012).

Biomarkers could be easily and noninvasively diagnostic measurements (Bostanci & Belibasakis, 2018; Giannobile et al., 2009) as allowing to monitor individual tissue level processes in diagnosis of periodontitis, as well as in personalized prevention, maintenance, and management of periodontal diseases (Bostanci et al., 2018). One of the most documented biomarkers associated with periodontal diseases is collagenase‐2 or matrix metalloproteinase‐8 (MMP‐8) (Bostanci et al., 2021; Gupta, Sahni, et al., 2021; Hernández et al., 2021a; Sorsa et al., 2006). MMP‐8 is released by polymorphonuclear leukocytes in its inactive latent proform, which is, however, converted to an active form during the progressive phase of periodontitis (Sorsa et al., 2016). Active MMP‐8 (aMMP‐8) can conveniently be measured with a(MMP)‐8 lateral flow point of care (POC) chairside test made from mouth rinse at oral health reception in 5 min providing up‐to‐date online and real‐time information (Alassiri et al., 2018; Heikkinen et al., 2016; Heikkinen, Pakbaznejad Esmaeili, et al., 2017; Heikkinen, Raivisto, et al., 2017; Heikkinen et al., 2019; Leppilahti et al., 2018; Nwhator et al., 2014; Räisänen et al., 2018, 2019; Raivisto, Heikkinen, et al., 2020; Sorsa et al., 1988)—already alarming initial signs of periodontitis (Heikkinen et al., 2016; Heikkinen, Pakbaznejad Esmaeili, et al., 2017; Heikkinen et al., 2019; Nwhator et al., 2014; Räisänen et al., 2019; Raivisto, Heikkinen, et al., 2020; Raivisto, Sorsa, et al., 2020). Notably, total MMP‐8 is not as accurate containing both latent and active forms of MMP‐8 enzymes (Sorsa et al., 2016). Furthermore, using this aMMP‐8‐chairside‐test with a mouth rinse, we get a more accurate test result than using the whole saliva, which contains many secretions, cells, microorganisms, products, and food debris (Bostanci et al., 2018, 2020; Kaufman & Lamster, 2000).

Revised periodontitis case definition system was published in 2018. It was to define periodontitis by its stages: the severity and extent of periodontal tissue destruction and complexity of management, as well as grades, for example, how promptly periodontitis proceeds (so‐called “staging and grading,” respectively; Tonetti et al., 2018). Diagnosis of periodontitis is based on both clinical and radiographic examinations as well as the clinical attachment level, bleeding on probing describing gingival inflammation, and risk‐related factors, such as smoking and diabetes, associated with this disease (Chapple et al., 2018). Clinical and radiographic information reflects events that have already occurred. Thus today, biomarkers, such as aMMP‐8 lateral‐flow chairside or point‐of‐care‐test (PoC‐test) results have also come to complement the overall picture of the patient's current situation in the dental reception.

We wish to clarify the usefulness of aMMP‐8 chairside‐test (aMMP‐8 POCT) results to alert the initial or early periodontitis in adolescents, a potential periodontitis risk group with a cut‐off of 20 ng/ml (Hoffmann et al., 2009) with respect to Stage I according to the current periodontitis classification system.

Our hypothesis is that

(1) aMMP‐8 levels above 20 ng/ml combined with other clinical measurements such as deep pockets (at least 4 mm) and >BOP 10% among adolescents have Stage I, initial or early periodontitis.
(2) aMMP‐8 levels above 20 ng/ml lacking with other clinical measurements such as deep pockets (at least 4 mm) and >BOP 10% among adolescents have a risk for Stage I, initial periodontitis.
(3) aMMP‐8 levels above 20 ng/ml combined with other clinical measurements such as deep pockets (at least 4 mm) and <BOP10% among adolescents have a risk for Stage I, initial periodontitis.
(4) aMMP‐8 levels above 20 ng/ml lacking with other clinical measurements such as deep pockets (at least 4 mm) and <BOP10% among adolescents have a risk for Stage I, initial periodontitis.
(5) aMMP‐8 levels below 20 ng/ml combined with other clinical measurements such as deep pockets (at least 4 mm) and >BOP10% among adolescents have a risk for Stage I, initial periodontitis.
(6) Adolescents with αMMP‐8 levels below 20 ng/ml and missing other clinical measurements, such as deep pockets (at least 4 mm) and BOP10%, have gingivitis.
(7) aMMP‐8 levels below 20 ng/ml combined with other clinical measurements such as deep pockets (at least 4 mm) and <BOP10% among adolescents have a risk for Stage I, initial periodontitis.
(8) aMMP‐8 levels below 20 ng/ml lacking with other clinical measurements such as deep pockets (at least 4 mm) and <BOP10% among adolescents are healthy.

As regards paragraphs 2 and 3, it is difficult to say in which direction the situation is evolving, thus these could be determined as, “subclinical stages the gray and reversible area,” from which it is thus still possible to “end up” or “return” periodontally healthy or diseased.

## 2 MATERIALS AND METHODS

Studies were carried out at the Kotka Health Center in Eastern Finland in 2014–2015 (Heikkinen, Raivisto, et al., 2017) and at the Hämeenlinna Health Center in Southern Finland in 2017–2018 (Kaufman & Lamster, 2000). Participants were 15–17 years old in the Kotka study and 14–15 years old in the Hämeenlinna study. All participants aged 14–17 years gave their informed consent for the study. This study has statements on ethical approval by the Ethics Committees of Kymenlaakso Regional Hospital and Ethics Committee of the Helsinki and Uusimaa Hospital District (HUS Dnro 260/13/03/00/13) and by the city of Hämeenlinna and the Ethics Committee of the Helsinki and Uusimaa Hospital District (HUS Dnro 1770/2017), Finland. Whole‐saliva samples were collected for 47 subjects in Kotka and mouth rinse samples were collected for 70 subjects in Hämeenlinna.

Lateral flow chairside test for aMMP‐8 was performed and analyzed by a method for chairside diagnostic test‐kit (aMMP‐8 POCT) based on the lateral flow immunochromatography principle (Hanemaaijer et al., 1997; Sorsa et al., 1999). Time‐resolved immunofluorometric assay was applied to determine salivary levels of aMMP‐8 by the use of monoclonal antibodies against aMMP‐8 (Gupta, Mohindra, et al., 2021; Hernández et al., 2021b; Umeizudike et al., 2022).

All participants were examined for caries and periodontal status for the full mouth. Periodontal pocket depth (PPD) (at least 4 mm) was measured for every tooth at four sites in the Kotka study and at six sites in the Hämeenlinna study. Bleeding on probing (BOP) values were examined for four sites for every tooth. Participants filled in a questionnaire that consisted of questions concerning health behavior such as oral hygiene habits, use of tobacco products, alcohol, and drug in Hämeenlinna as well as in the Kotka study.

Stage I periodontitis signifies maximum probing depth ≤4 mm with interdental Clinical Attachment Loss (CAL) at site of greatest loss of 1 to 2mm (Papapanou et al., 2018). In its early phase, periodontitis has no remarkable visible signs in clinical or radiological examination (Heikkinen,  Pakbaznejad Esmaeili, et al., 2017). Subclinical periodontitis could be situated between gingivitis and Stage I periodontitis, as a gray area. This state may turn into Stage I, gingivitis or heathy (Raivisto,Sorsa, et al., 2020).

## 3 RESULTS

Of the participants, who did not have any deepened periodontal pockets, 18 were test negatives. Of the participants who had at least one 4 mm deep periodontal pocket, 15 were test negatives and 14 test positives. The association between PPD, BOP%, and aMMP‐8 point‐of‐care‐test result is summarized in Table 1. No associations between BOP% and aMMP‐8 were observed with or without adjusting for PPD.

**Table 1:** The association between PPD, BOP%, and aMMP‐8 POCT results

PPD	BOP%	aMMP‐8 POCT result	*N*	*p *value
No PPD	BOP < 10	Test negative	57	.6532
		Test positive	20	
	BOP ≥ 10	Test negative	4	
		Test positive	2	
PPD ≥ 1	BOP < 10	Test negative	8	1.000
		Test positive	7	
	BOP ≥ 10	Test negative	10	
		Test positive	9	
Total	BOP < 10	Test negative	65	.2279
		Test positive	27	
	BOP ≥ 10	Test negative	14	
		Test positive	11	

Abbreviations: BOP%, bleeding on probing percentage; *N*, number of adolescents; PPD, periodontal probing depth, one or more ≥4mm deepened periodontal pocket(s).

*p* values calculated by Fisher's exact test.

Of all 117 participants, *N* = 38 (32.5%) was aMMP‐8 POCT positive, and *N* = 34 (29.1%) had at least one 4 mm periodontal deepened pocket; however, only *N* = 16 (13.7%) had both characteristics, if not considered bleeding on probing. None in the gingivitis group had periodontal deepened pockets and the aMMP‐8 level was less than 20 ng/ml. Instead, all in the Stage I group had periodontal deepened pockets as well as aMMP‐8 level was more than 20 ng/ml (Table 2).

**Table 2:** Grading of the risk of disease progression in health, gingivitis, subclinical (=subclin) stage,^a^ and Stage I (initial periodontitis) by aMMP‐8, total *N* = 117

Indicators of active periodontal tissue destruction	Total N	Healthy *N* = 57 (15^b^, 42^c^) 48%	Gingivitis *N* = 4 (3^b^, 1^c^) 3%	Subclin^a^ stage, *N* = 20 (0^b^, 20^c^)	Subclin^a^ stage, *N* = 2 (0^b^, 2^c^)	Subclin^a^ stage, *N* = 7 (5^b^, 2^c^)	Subclin^a^ stage, *N* = 8 (6^b^, 2^c^)	Subclin^a^ stage, *N* = 10 (9^b^, 1^c^)	Stage I *N* = 9 (9^b^, 0^c^) 8%
	PPD ≥ t 4 mm	No	No	No	No	Yes	Yes	Yes	Yes
	aMMP‐8 level^d^	<20 ng/ml	<20 ng/ml	>20 ng/ml	>20 ng/ml	>20 ng/ml	<20 ng/ml	<20 ng/ml	>20 ng/ml
	BOP ≥ 10%	No	Yes	No	Yes	No	No	Yes	Yes

Abbreviations: BOP, bleeding on probing; PPD, periodontal probing depth.

^a^ The subclinical (=subclin) stage (“gray area”) could not be classified either as gingivitis or Stage I; thus, it is named the subclinical stage.

^b^ Kotka study participants, 15–16 years old.

^c^ Hämeenlinna study participants, 14 years old.

^d^ Positive (at least 20 ng/ml) or negative (<20 ng/ml).

Of all 70 participants in the Jukola study, 24 (34.3%) were aMMP‐8 chairside test positive. Of the participants, who did not have deepened periodontal pockets, 43 were test negatives and 22 test positives. Of the participants who had at least one 4 mm deep periodontal pocket, three were test negatives and two test positives. Whereas in Kotka study, among 47 participants, 14 (29.2%) were aMMP‐8 chairside test positive.

In all almost half of participants were classified to be healthy, only 3% had gingivitis and 8% Stage I, respectively. The subclinical stage (“gray area”) *N* = 47 (40,2%) could not be classified either as gingivitis or Stage I. Of them *N* = 29 (60,2%) were aMMP‐8 POCT test positive.

## 4 DISCUSSION

Our study revealed that the presence of periodontal deep pockets (probing depth at least 4 mm) with positive aMMP‐8‐test result (above 20 ng/ml) eventually indicated active periodontal tissue degeneraftion. Notable, almost half of the participants were healthy, and only 3% suffered from gingivitis. Furthermore, there were young individuals, in our “subclinical” group (40%), who could not be classified to be as periodontally healthy or diseased (nor gingivitis or Stage I periodontitis). However, all these adolescents are eventual risk patients requiring proper individual and personalized intervention prevention in the oral healthcare settings according to their periodontal status and health behavior (Heikkinen et al., 2016; Heikkinen, Pakbaznejad Esmaeili, et al., 2017; Heikkinen, Raivisto, et al., 2017; Heikkinen et al., 2019; Räisänen et al., 2019). We argue that in the initial or subclinical stage—as well as in gingivitis, periodontal health could turn into either a healthy or diseased state depending on oral health behavior and habits, such as smoking and tooth brushing, as well given preventive intervention provided by the oral health professionals. We also assume that “active initial degeneration” could actually be defined as “subclinical periodontitis or pre‐periodontitis.”

Silbereisen et al. (2020) published an experimental study on gingival inflammation. They observed that western blot analysis could not detect active MMP‐8 (aMMP‐8) forms and species during the induction and resolution of gingival inflammation, which is an important finding to help us to understand the possible the mechanisms that convert marginal gingivitis to periodontitis (Silbereisen et al., 2020). Furthermore, they observed, that compared to periodontitis, MMP‐8 is not activated, that is, converted to active forms and related fragmented low molecular size species throughout the course of gingival inflammation induction and resolution (Silbereisen et al., 2020). This is in agreement and further expands the findings revealing that MMP‐8 is not activated in gingivitis (Romanelli et al., 1999). In Kotka “initial periodontitis in adolescents”‐study and in “chronic periodontitis in adults‐study” of Nwhator et al. (2014) the cut‐off was 20 ng/ml for chairside‐ (aMMP‐8 POCT) tests (Heikkinen et al., 2016; Nwhator et al., 2014). To be pointed, that no research results have been published using other cut‐offs, such as cut off 10 ng/ml for aMMP‐8 values. However, recently Deng et al. (2021) have presented that “according to the manufacturer's recommendation, the aMMP‐8 level was considered in the healthy range (concentration ≤10 ng/ml); as active periodontal degeneration (10 ng/ml <concentration ≤20 ng/ml); or inflammatory tissue destruction (concentration >20 ng/ml), respectively.” There is no evidence supporting this classification data: healthy, active periodontal degeneration and inflammatory tissue destruction with given such aMMP‐8 levels. To our knowledge it has not come to light that aMMP‐8 POCTs are not available other than with cut‐off of 20 ng/ml (Deng et al., 2021; Hoffmann et al., 2009; Nwhator et al., 2014; Sorsa et al., 1988).

Recently, regarding chronic adult periodontitis, two distinct studies addressing the implementation of an aMMP‐8 chairside test as the key chairside biomarker in the new staging and grading classification of periodontitis have yielded varied results (Sorsa et al. 2020, 2021). Careful comparison of the clinical status and data of the patient materials involved in these two cohort studies revealed that  Deng et al.'s (2021) cohort exerted clearly lesser gingivitis cases with lower Gingiva Index (GI) and, that is, with less deep PPD and less increased BOP% values (Deng et al., 2021) than the ones in the Sorsa et al.'s cohort (Sorsa et al., 2020). This could be explained by the recorded differences in the sensitivities regarding the MMP‐8 POCT (Romanelli et al., 1999). A recent study revealed, that aMMP‐8 POCT with cut‐off of 20 ng/ml, but not total MMP‐8 levels, and related to oral microbial dysbiosis of periodontal pathogenesis proteolysis, can be linked to new staging and grading classification (Bostanci et al., 2021; Gupta, Sahni, et al., 2021).

The present study involves adolescents who are “pure,” untreated, and without a long treatment history. This is the strength of our research as well as the fact that there are not many or any studies on the oral health of children and adolescents. We observed that no significant association between BOP% and aMMP‐8 POCT result with or without adjusting for PPD. This is in line with Räisänen et al. (2021) as they pointed out that BOP levels mainly could be described as an important indicator of the extent of the bacterial challenge and its adverse effects on the gingival inflammation (Räisänen et al., 2021). As, for example, Lee et al. (1995) have demonstrated in their prospective study, elevated aMMP‐8 levels have a direct role in the pathological destruction of periodontal connective tissue (Lee et al., 1995). Furthermore, detection of BOP by probing is prone to errors (Bostanci et al., 2019; Karayiannis et al., 1992). Thus, relying on BOP levels (below 10% or 20%) may provide insufficient information about the periodontal treatment need of an adolescent depending on his/her level of oral hygiene.”

The limitation of this study is the lack of clinical attachment loss (CAL). However, PPD and CAL are observed to be linked to each other, and enhanced CALs are reflected as deepened periodontal pockets (Badersten et al., 1990; Claffey & Egelberg, 1994). It is good to remember that radiological examinations are not possible due to the examination of adolescents alone from a research perspective to elucidate the relationship between CAL and periodontal deepened pocket. One may also consider whether CAL is relevant to adolescents because they have only minimal periodontal history behind them (Heikkinen, Pakbaznejad Esmaeili, et al., 2017). It is worth remembering that clinical and radiological findings with respect to early signs of periodontitis may not be compatible (Ziebolz et al., 2011) as well as that early periodontal bone loss in adolescents the impact of subjective criteria on assessment could lead to different interpretations of periodontal condition (Jenkins et al., 1992). Furthermore, Jenkins et al. (1992) pointed out that “it is important to establish the effect that beam angulation and amelo‐cemental junction (ACJ) morphology may have on the radiographic appearance of this structure.” Notable, according to a retrospective study by Thorbert‐Mros et al. (2017), where radiographs were evaluated from patients with generalized, severe periodontitis, they reported according to their study, that onset of disease is occurred on the average between 22.3 and 28.1 years of age. They also pointed that sites exhibiting severe bone loss were detected at the age of about 32.4 years (Thorbert‐Mros et al., 2017). Furthermore, Leppilahti et al. (2018) demonstrated that aMMP‐8 POCT preceded periodontal hard tissue destruction in African young (aged 18–22 years) females (Räisänen et al., 2019). Additionally, the relatively small size of the data could be regarded as a limitation of this study. Therefore, more research on initial periodontitis among adolescents is essentially needed in the future to possible confirm and further extend our results.

All our Finnish adolescent cohorts (both Kotka and Hämeenlinna studies) were systemically healthy and nearly half of all were periodontally healthy patients with healthy native periodontium completely lacked any periodontal disease experience and signs, thus forming the healthy controls. The age‐ and sex‐matched subjects with one at least 4 mm deep periodontal pocket formed the initial or early periodontitis patient group. These patients and their healthy controls completely lacked any advanced periodontal disease experience and did not exert any systemic predisposing diseases or other risk factors such as smoking. In the present study such adolescents with at least one 4 mm deep pocket could be conveniently real‐time picked up with help by an aMMP‐8 chairside test in two cases of three if an account is taken of cases with active cases too. It has been established that all deepened periodontal pockets are not in the clinically active disease phase, and thus such deepened periodontal pockets may not exert elevated aMMP‐8 levels (Silbereisen et al., 2020). This could explain, at least partly, why aMMP‐8 negative deepened pockets in fact, observed too, eventually represent clinical disease inactive sites.

Elevated (>20 ng/ml), these positive aMMP‐8 POCT results, can be regarded as initial alarmer such emerging risk for periodontitis. Eventually they may represent pre‐periodontitis cases similarly to prediabetes regarding to diabetic diseases. In this paper we propose and summarize for the disease classification: especially for the young people, it is important to take a closer look at the classification and accordingly refine it, who are healthy, and who are not. It is important to consider all groups at potential risk of developing periodontitis. And, of course, target the intervention or preventive treatment to those at the Stage I. Stage I as well as subclinical stage are preceded by antecedent stages that should be tackled by oral health care prevention and personalized treatment modalities by professionals. Overall, this should be utilized in the preventive personalized interventions by oral health professionals. This will be a saving in the future.

## AUTHOR CONTRIBUTIONS

Anna Maria Heikkinen performed and planned the whole study, collected the study of Kotka, wrote and approved the manuscript. Teija Raivisto, planned and collected the study of Hämeenlinna, wrote and approved the manuscript. Ismo Räisänen analyzed the data and approved the manuscript. Taina Tervahartiala analyzed the data and approved the manuscript. Nagihan Bostanci participated in the design and writing of the article and approved the manuscript. Timo Sorsa participated in the design, analyzing and writing of the article and approved the manuscript.

## CONFLICTS OF INTEREST

Timo Sorsa is an inventor of US patent numbers of 5652223, 5736341, 5866432, 6143476, 20170023571A1 (granted 6.6.2019), WO 2018/060553 A1 (granted 31.5.2018), 10 488 415 B2, and a Japanese patent 2016‐554676. The remaining authors declare no conflict of interest.

## Data Availability

Data are available on request from the authors. The data that support the findings of this study are available from the corresponding author upon reasonable request.
